# Supplementary figures and images for: Theory on the Dynamics of Oscillatory Loops in the Transcription Factor Networks
Source: PLoS One. 2014 Aug 11;9(8):e104328. doi: 10.1371/journal.pone.0104328 (PMC4128676; doi:10.1371/journal.pone.0104328)

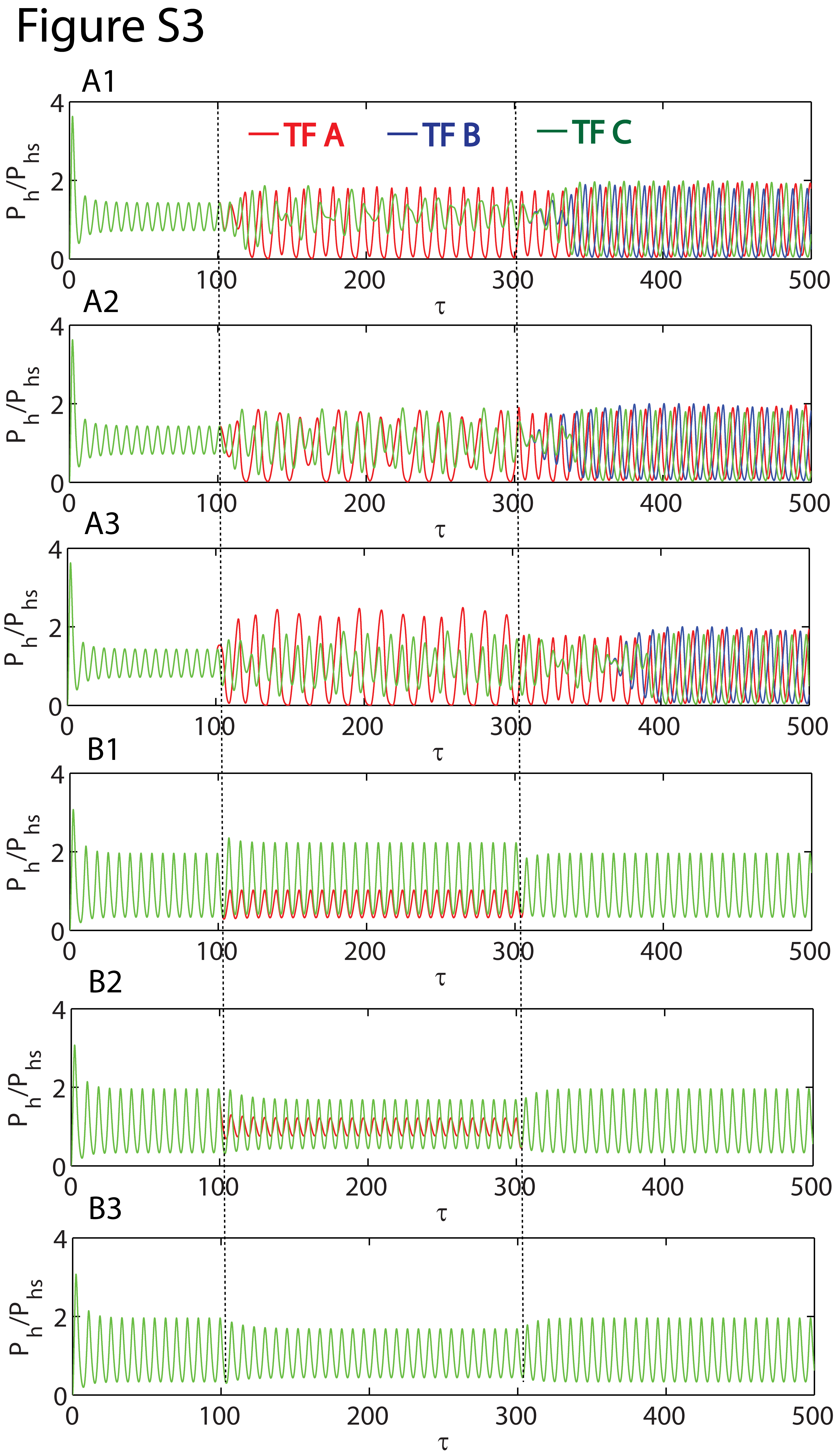

Supplement: File S1 — This file contains Figure S1-Figure S5. Figure S1. Three gene repressilator model. A1-4. Phase portraits and trajectories of TF genes A, B and C of a repressilator. Simulation settings are , , and which required a critical Hill coefficient of Cna = 2. Total simulation time is 200 (number of lifetimes of the protein product of TF gene A) and integration step is . To trigger the oscillations, we have introduced the asymmetry in the initial condition for the promoter state occupancy of TF gene A as . Oscillations starts with a time delay whose value depends of the magnitude of this disproportion in the parameter values. A5. Roots of the twelfth degree characteristic polynomial associated with the Jacobian matrix of Eqs (26) for settings given in A1. B1-2. Effects of perturbation in that is raised to ( in B2) in the time interval from 0 to 100. Increase in increases the period of oscillations of the entire system from to 24.5 and reduces the amplitudes of TF genes A and C. The amplitudes of TF genes A/B/C are such that A<C<B. B3-4. Effects of perturbation in which are raised to in the time interval from 0 to 100. Increase in increases the period of oscillation of the entire system from to 30 and reduces the amplitudes of TF genes A and B and increases the amplitude of C and the amplitudes of TF genes are such that B<A<C (B3). Increase in increases the period of oscillation of the entire system as in B3 where the amplitudes of TF genes A/B/C are such that B<A<C (B4). Figure S2. Dynamics of three independent Goodwin-Griffith oscillators cyclically coupled. A1-3. Phase portraits of TF genes A/B/C which are three independent GG oscillators cyclically coupled through -OR- type logic as given in Figure 2C2 (without dashed lines). Simulation settings are , , and which required a critical Hill coefficient of Cna = 5 (we have set this to 6 for clarity of results). Total simulation time is 500 (number of lifetimes of the protein product of TF gene A) and integration step is . In [file pone.0104328.s001.zip › FIGURE S3.tif]

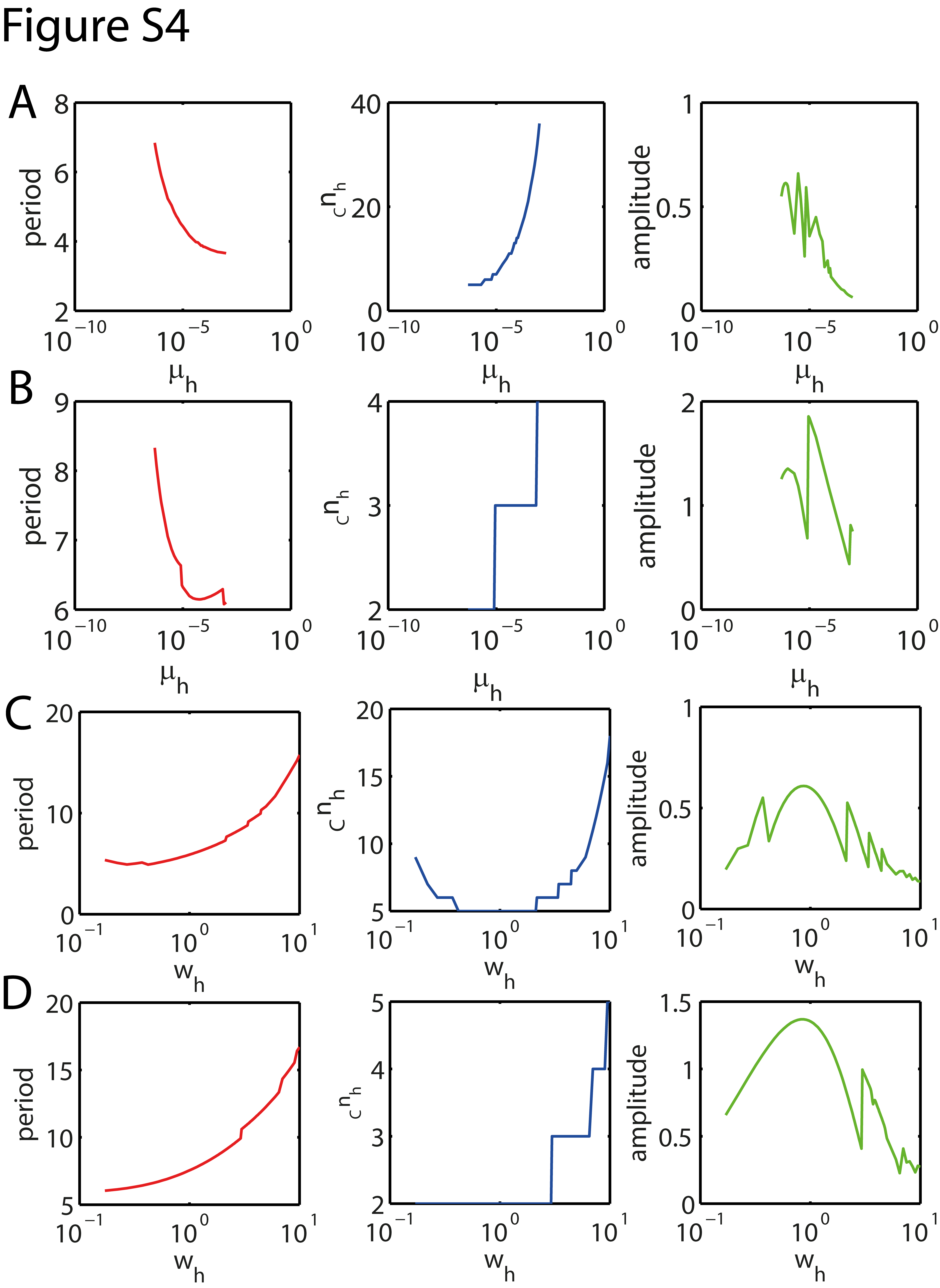

Supplement: File S1 — This file contains Figure S1-Figure S5. Figure S1. Three gene repressilator model. A1-4. Phase portraits and trajectories of TF genes A, B and C of a repressilator. Simulation settings are , , and which required a critical Hill coefficient of Cna = 2. Total simulation time is 200 (number of lifetimes of the protein product of TF gene A) and integration step is . To trigger the oscillations, we have introduced the asymmetry in the initial condition for the promoter state occupancy of TF gene A as . Oscillations starts with a time delay whose value depends of the magnitude of this disproportion in the parameter values. A5. Roots of the twelfth degree characteristic polynomial associated with the Jacobian matrix of Eqs (26) for settings given in A1. B1-2. Effects of perturbation in that is raised to ( in B2) in the time interval from 0 to 100. Increase in increases the period of oscillations of the entire system from to 24.5 and reduces the amplitudes of TF genes A and C. The amplitudes of TF genes A/B/C are such that A<C<B. B3-4. Effects of perturbation in which are raised to in the time interval from 0 to 100. Increase in increases the period of oscillation of the entire system from to 30 and reduces the amplitudes of TF genes A and B and increases the amplitude of C and the amplitudes of TF genes are such that B<A<C (B3). Increase in increases the period of oscillation of the entire system as in B3 where the amplitudes of TF genes A/B/C are such that B<A<C (B4). Figure S2. Dynamics of three independent Goodwin-Griffith oscillators cyclically coupled. A1-3. Phase portraits of TF genes A/B/C which are three independent GG oscillators cyclically coupled through -OR- type logic as given in Figure 2C2 (without dashed lines). Simulation settings are , , and which required a critical Hill coefficient of Cna = 5 (we have set this to 6 for clarity of results). Total simulation time is 500 (number of lifetimes of the protein product of TF gene A) and integration step is . In [file pone.0104328.s001.zip › FIGURE S4.tif]

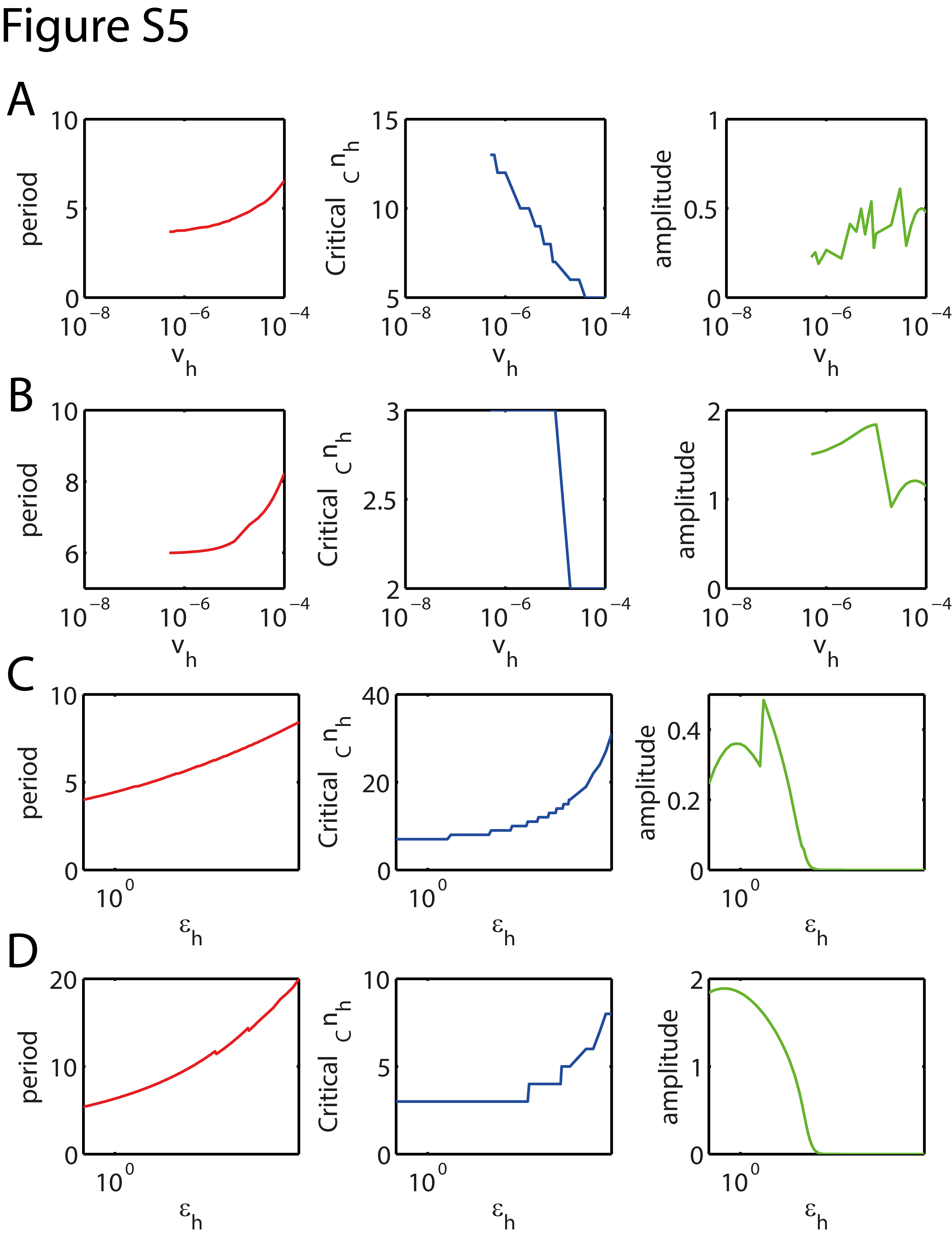

Supplement: File S1 — This file contains Figure S1-Figure S5. Figure S1. Three gene repressilator model. A1-4. Phase portraits and trajectories of TF genes A, B and C of a repressilator. Simulation settings are , , and which required a critical Hill coefficient of Cna = 2. Total simulation time is 200 (number of lifetimes of the protein product of TF gene A) and integration step is . To trigger the oscillations, we have introduced the asymmetry in the initial condition for the promoter state occupancy of TF gene A as . Oscillations starts with a time delay whose value depends of the magnitude of this disproportion in the parameter values. A5. Roots of the twelfth degree characteristic polynomial associated with the Jacobian matrix of Eqs (26) for settings given in A1. B1-2. Effects of perturbation in that is raised to ( in B2) in the time interval from 0 to 100. Increase in increases the period of oscillations of the entire system from to 24.5 and reduces the amplitudes of TF genes A and C. The amplitudes of TF genes A/B/C are such that A<C<B. B3-4. Effects of perturbation in which are raised to in the time interval from 0 to 100. Increase in increases the period of oscillation of the entire system from to 30 and reduces the amplitudes of TF genes A and B and increases the amplitude of C and the amplitudes of TF genes are such that B<A<C (B3). Increase in increases the period of oscillation of the entire system as in B3 where the amplitudes of TF genes A/B/C are such that B<A<C (B4). Figure S2. Dynamics of three independent Goodwin-Griffith oscillators cyclically coupled. A1-3. Phase portraits of TF genes A/B/C which are three independent GG oscillators cyclically coupled through -OR- type logic as given in Figure 2C2 (without dashed lines). Simulation settings are , , and which required a critical Hill coefficient of Cna = 5 (we have set this to 6 for clarity of results). Total simulation time is 500 (number of lifetimes of the protein product of TF gene A) and integration step is . In [file pone.0104328.s001.zip › FIGURE S5.tif]

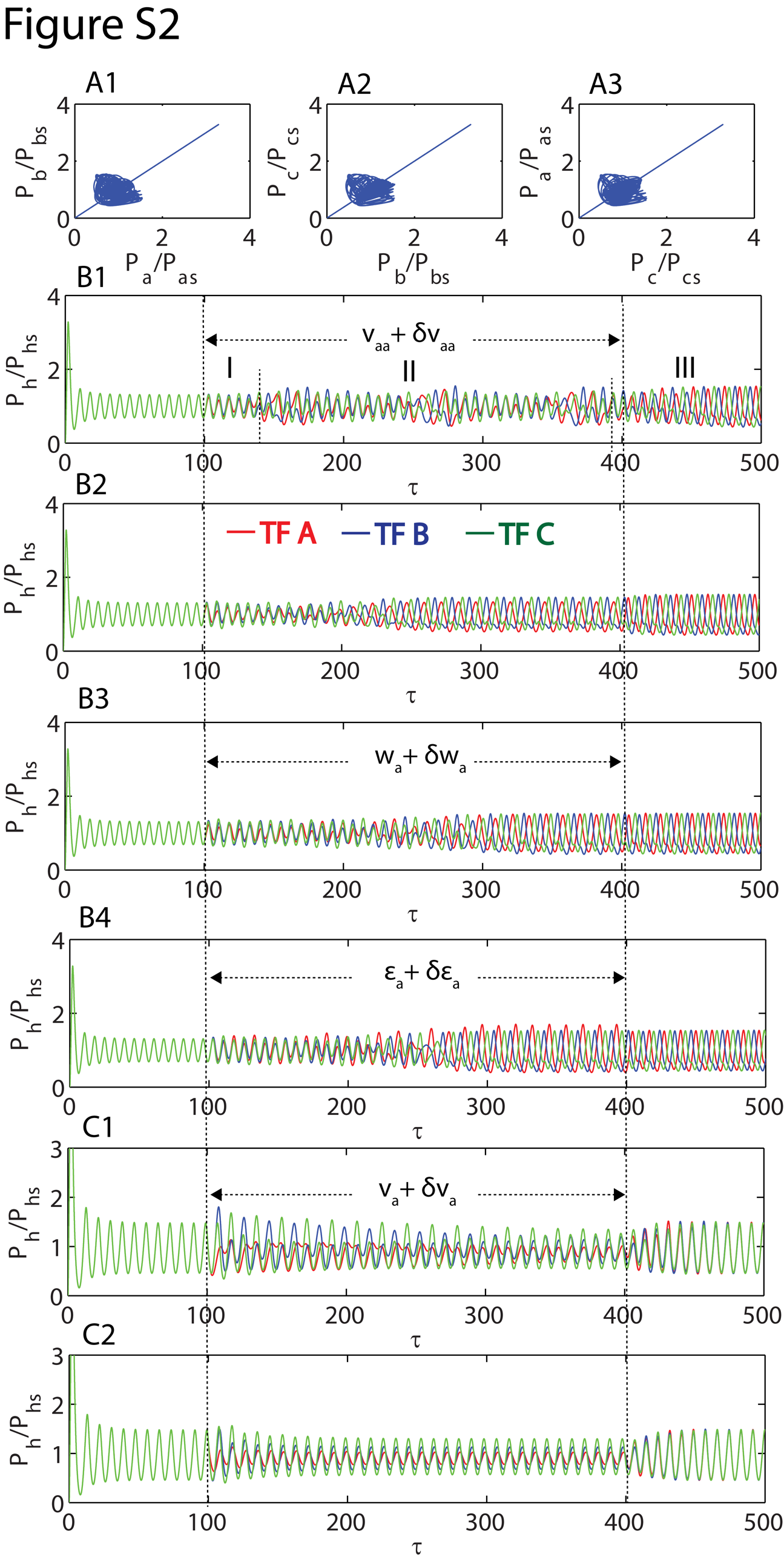

Supplement: File S1 — This file contains Figure S1-Figure S5. Figure S1. Three gene repressilator model. A1-4. Phase portraits and trajectories of TF genes A, B and C of a repressilator. Simulation settings are , , and which required a critical Hill coefficient of Cna = 2. Total simulation time is 200 (number of lifetimes of the protein product of TF gene A) and integration step is . To trigger the oscillations, we have introduced the asymmetry in the initial condition for the promoter state occupancy of TF gene A as . Oscillations starts with a time delay whose value depends of the magnitude of this disproportion in the parameter values. A5. Roots of the twelfth degree characteristic polynomial associated with the Jacobian matrix of Eqs (26) for settings given in A1. B1-2. Effects of perturbation in that is raised to ( in B2) in the time interval from 0 to 100. Increase in increases the period of oscillations of the entire system from to 24.5 and reduces the amplitudes of TF genes A and C. The amplitudes of TF genes A/B/C are such that A<C<B. B3-4. Effects of perturbation in which are raised to in the time interval from 0 to 100. Increase in increases the period of oscillation of the entire system from to 30 and reduces the amplitudes of TF genes A and B and increases the amplitude of C and the amplitudes of TF genes are such that B<A<C (B3). Increase in increases the period of oscillation of the entire system as in B3 where the amplitudes of TF genes A/B/C are such that B<A<C (B4). Figure S2. Dynamics of three independent Goodwin-Griffith oscillators cyclically coupled. A1-3. Phase portraits of TF genes A/B/C which are three independent GG oscillators cyclically coupled through -OR- type logic as given in Figure 2C2 (without dashed lines). Simulation settings are , , and which required a critical Hill coefficient of Cna = 5 (we have set this to 6 for clarity of results). Total simulation time is 500 (number of lifetimes of the protein product of TF gene A) and integration step is . In [file pone.0104328.s001.zip › FIGURE S2.tif]
